# Occupational Exposure to Pesticides and the Incidence of Lung Cancer in the Agricultural Health Study

**DOI:** 10.1289/EHP456

**Published:** 2016-07-06

**Authors:** Matthew R. Bonner, Laura E. Beane Freeman, Jane A. Hoppin, Stella Koutros, Dale P. Sandler, Charles F. Lynch, Cynthia J. Hines, Kent Thomas, Aaron Blair, Michael C.R. Alavanja

**Affiliations:** 1 Department of Epidemiology and Environmental Health, School of Public Health and Health Professions, University at Buffalo, Buffalo, New York, USA; 2 Occupational and Environmental Epidemiology Branch, Division of Cancer Epidemiology and Genetics, National Cancer Institute, National Institutes of Health (NIH), Department of Health and Human Services (DHHS), Bethesda, Maryland, USA; 3 Department of Biological Sciences, Center for Human Health and the Environment, North Carolina State University, Raleigh, North Carolina, USA; 4 Epidemiology Branch, National Institute of Environmental Health Sciences, NIH, DHHS, Research Triangle Park, North Carolina, USA; 5 College of Public Health, University of Iowa, Iowa City, Iowa, USA; 6 Division of Surveillance, Hazard Evaluations, and Field Studies, National Institute for Occupational Safety and Health, Cincinnati, Ohio, USA; 7 National Exposure Research Laboratory, U.S. Environmental Protection Agency, Research Triangle Park, North Carolina, USA

## Abstract

**Background:**

Occupational pesticide use is associated with lung cancer in some, but not all, epidemiologic studies. In the Agricultural Health Study (AHS), we previously reported positive associations between several pesticides and lung cancer incidence.

**Objective:**

We evaluated use of 43 pesticides and 654 lung cancer cases after 10 years of additional follow-up in the AHS, a prospective cohort study comprising 57,310 pesticide applicators from Iowa and North Carolina.

**Methods:**

Information about lifetime pesticide use and other factors was ascertained at enrollment (1993–1997) and updated with a follow-up questionnaire (1999–2005). Cox proportional hazards models were used to calculate hazard ratios (HRs) and 95% confidence intervals (CIs), adjusting for smoking (smoking status and pack-years), sex, and lifetime days of use of any pesticides.

**Results:**

Hazard ratios were elevated in the highest exposure category of lifetime days of use for pendimethalin (1.50; 95% CI: 0.98, 2.31), dieldrin (1.93; 95% CI: 0.70, 5.30), and chlorimuron ethyl (1.74; 95% CI: 1.02, 2.96), although monotonic exposure–response gradients were not evident. The HRs for intensity-weighted lifetime days of use of these pesticides were similar. For parathion, the trend was statistically significant for intensity-weighted lifetime days (*p* = 0.049) and borderline for lifetime days (*p* = 0.073). None of the remaining pesticides evaluated was associated with lung cancer incidence.

**Conclusions:**

These analyses provide additional evidence for an association between pendimethalin, dieldrin, and parathion use and lung cancer risk. We found an association between chlorimuron ethyl, a herbicide introduced in 1986, and lung cancer that has not been previously reported. Continued follow-up is warranted.

**Citation:**

Bonner MR, Beane Freeman LE, Hoppin JA, Koutros S, Sandler DP, Lynch CF, Hines CJ, Thomas K, Blair A, Alavanja MCR. 2017. Occupational exposure to pesticides and the incidence of lung cancer in the Agricultural Health Study. Environ Health Perspect 125:544–551; http://dx.doi.org/10.1289/EHP456

## Introduction

Lung cancer is the leading cause of cancer-related death in the United States (American Cancer Society 2017) and in the world (Torre et al. 2015). Lung cancer mortality and incidence is lower among farmers in the United States than among the general population (Blair et al. 1993; Blair and Freeman 2009) potentially because of the low prevalence of smoking among U.S. farmers (Alavanja et al. 2004; Blair et al. 1992). Nonetheless, increased lung cancer mortality among licensed pesticide applicators has been reported (Barthel 1981; Becher et al. 1996; Blair et al. 1983; MacMahon et al. 1988; Pesatori et al. 1994), raising the possibility that exposure to certain pesticides may increase the risk of lung cancer among farmers. Only a few epidemiologic studies have assessed exposure to specific pesticides (Austin et al. 1989; MacMahon et al. 1988; Pesatori et al. 1994). MacMahon et al. (1988) reported a slight increase in the lung cancer standardized mortality ratio (SMR) [SMR = 135; 90% confidence interval (CI): 114, 158] among pesticide applicators and termite control operators exposed to chlordane and heptachlor. Blair et al. (1983) also observed an excess of lung cancer among termite and other structural pest–control applicators. Using banked serum samples from 919 residents of Charleston, South Carolina, Austin et al. (1989) did not find an association between serum DDT levels and respiratory cancer mortality among 19 cases. In a small, nested case–control study of structural pesticide workers in Florida, Pesatori et al. (1994) observed suggestive positive associations for diazinon [odds ratio (OR) = 2.0; 95% CI: 0.7, 5.5], carbaryl (OR = 4.2; 95% CI: 0.6, 27.2), dichlorodiphenyltrichloroethane (DDT) (OR = 2.6; 95% CI: 0.5, 14.3), and propoxur (OR = 12.4; 95% CI: 1.05, 100.3); no associations were observed for malathion, chlorpyrifos, parathion, or chlordane. We previously reported positive associations between select pesticides and the occurrence of lung cancer in the Agricultural Health Study (AHS) (Alavanja et al. 2004). Of the 50 pesticides evaluated, 7 (dicamba, metolachlor, pendimethalin, carbofuran, chlorpyrifos, diazinon, and dieldrin) showed some evidence of positive associations with lung cancer incidence. Pesticide-specific analyses of diazinon (Jones et al. 2015) and metolachlor (Silver et al. 2015) that evaluated lung cancer risk, among other cancer sites, have recently been published from the AHS. Jones et al. (2015) reported increased lung cancer incidence among male pesticide applicators with the highest exposure category of lifetime days of diazinon use [rate ratio (RR) = 1.60; 95% CI: 1.11, 2.31; *p*_trend_ = 0.02] as well as with intensity-weighted lifetime days of diazinon use (RR=1.41; 95% CI: 0.98, 2.04; *p*_trend_ = 0.08). Silver et al. (2015) found no association with either lifetime days or intensity-weighted lifetime days of metolachlor use.

Herein, we have used the AHS to investigate associations between lifetime use of 43 pesticides and the incidence of lung cancer with an additional 414 lung cancer cases and 10 years of follow-up beyond an earlier evaluation (Alavanja et al. 2004) and with updated information regarding more recent pesticide use and cigarette smoking status.

## Methods and Materials

The AHS has been described previously (Alavanja et al. 1996). Briefly, we enrolled 57,310 restricted-use pesticide applicators residing in Iowa [commercial and private (farmer) = 36,792] and North Carolina (private applicators = 20,518) between 1993 and 1997 (AHS data release: P1REL201209.00, P2201209.00, and AHSREL201304.01). Vital status through 31 December 2011 was ascertained via linkage with state mortality files and the National Death Index. First primary, incident lung cancer cases that occurred between enrollment and 31 December 2010 in North Carolina and 31 December 2011 in Iowa were identified via linkage with the Iowa and North Carolina state cancer registries. Prevalent cancers (n = 1,094) and individuals who sought to obtain pesticide registration in Iowa or North Carolina but did not reside in these states (n = 341) were excluded from the analysis. Participants (n = 1,113) who moved out of Iowa or North Carolina were censored at the year they departed. All applicable Institutional Review Boards approved the protocol, and all participants provided informed consent.

### Exposure Assessment

At enrollment, participants completed a self-administered questionnaire (http://aghealth.nih.gov/collaboration/questionnaires.html) indicating whether they had ever mixed or applied 50 specific pesticides. The number of years and the number of days per year the applicator personally mixed or applied a particular pesticide was also queried on the enrollment questionnaire for 22 pesticides. This detailed information about days and years of use for the remaining 28 pesticides was obtained in a supplementary take-home questionnaire completed by ~44% of the cohort. In addition, the enrollment questionnaire gathered information on pesticide application methods, mixing, repair of pesticide application equipment, and the use of personal protective equipment (PPE). Smoking history, alcohol consumption in the past 12 months, fruit and vegetable consumption, other agricultural activities, non-farm occupational exposures, family history of cancer, medical conditions, and medicines were also ascertained at enrollment. Blair and colleagues have previously shown that the reliability of reporting of pesticide use in the AHS questionnaire is similar to that for other factors routinely obtained by questionnaire for epidemiologic studies (Blair et al. 2002).

Lifetime exposure-days of use for each of the 50 pesticides was calculated from the questionnaire data as the product of the number of years a participant personally mixed or applied each specific pesticide times the number of days in an average year that pesticide was used. In addition, we used an estimate of exposure intensity based on an algorithm generated by Dosemeci et al. (2002) that was developed from a comprehensive review of the literature and was updated and supplemented by Coble et al. (2011). This algorithm also used pesticide monitoring conducted in the AHS (Hines et al. 2008; Thomas et al. 2010) to calculate an intensity-weighted lifetime exposure-days score for each pesticide [exposure intensity × lifetime exposure-days]. The exposure intensity score weights aspects of pesticide use that may modify the intensity of exposure, such as whether an applicator personally mixed pesticides for application, application methods used, repair of pesticide application equipment, and the use of PPE. Dermal absorption is generally considered the major route of exposure for many pesticides (Maroni et al. 2000). Pesticide monitoring in the AHS found that chemical-resistant glove use was a more important determinant of urinary, airborne and dermal levels of pesticides than was initially assumed (Hines et al. 2008; Thomas et al. 2010). Consequently, the updated exposure intensity score weighted the use of protective gloves more heavily (Coble et al. 2011).

Information on pesticide use was updated between 1999 and 2005 with the use of a computer-assisted telephone interview (CATI). Participants were asked to report all pesticides used in the year prior to the interview as well as the frequency of use. Because only 36,342 applicators (63%) completed both the baseline and follow-up questionnaires, we used multiple imputation with logistic regression and stratified sampling to impute missing pesticide exposure information for 20,968 applicators who did not complete the follow-up interview (Heltshe et al. 2012).

In addition to updating pesticide use information between 1999 and 2005 with the CATI, smoking status (current, past, never), but not pack-years, was also updated. To update pack-years of cigarette smoking among current smokers (*n* = 7,637), we multiplied the number of cigarettes smoked that was reported in the enrollment questionnaire by the number of intervening years between the enrollment and the follow-up interview. These additional pack-years of cigarette smoking were then added to the total pack-years calculated from the enrollment questionnaire to update total pack-years of cigarette smoking. For participants who reported being current smokers on the enrollment questionnaire but reported being former smokers in the follow-up interview (*n* = 1,712), the number of cigarettes smoked per day reported at enrollment was used in the aforementioned calculation, and the number of years of smoking during the intervening time period was estimated to comprise half the time period. This same algorithm was used for participants who reported being former smokers at enrollment, but reported smoking currently in the follow-up interview (*n* = 573). For participants missing information on smoking on the enrollment and the follow-up interview (*n* = 1,051), pack-years of smoking was not imputed. Similarly, because a small proportion of participants (*n* = 1,033) was missing information on the number of cigarettes smoked per day (enrollment questionnaire), pack-years of smoking was not imputed. In addition, participants missing information on other potential confounders (e.g., age, sex, total lifetime pesticide days) (*n* = 4,338) were also excluded. In total, 7,498 participants were excluded, leaving 49,812 (89%) participants for the statistical analysis of pesticide exposure.

### Statistical Analyses

We used Cox proportional hazards to estimate hazard ratios and 95% confidence intervals, using age at risk as the time scale, to assess potential associations between pesticide use and the incidence of lung cancer. We evaluated 43 specific pesticides here. Diazinon (Jones et al. 2015) and metolachlor (Silver et al. 2015) were not evaluated because results from the evaluation of these pesticides have recently been published. In addition, 5 other pesticides (trichlorfon, carbon tetrachloride/carbon disulfide, aluminum phosphide, ziram, and 2-(2,4,5-trichlorophenoxy)propionic acid (2,4,5-TP; fenoprop) were not evaluated because there were fewer than 15 exposed lung cancer cases, which is too few for meaningful analyses. Lifetime days of exposure and intensity-weighted lifetime exposure-days were both categorized into quartiles based on the distribution among the lung cancer cases to assess exposure–response gradients where possible. For 7 pesticides (aldrin, captan, carbofuran, coumaphos, dieldrin, heptachlor, and toxaphene) tertiles were used because of the relatively small number of exposed lung cancer cases. In addition to assessing cumulative lifetime exposure-days, we also conducted analyses in which lifetime exposure-days were lagged 5 and 15 years.

*A priori* covariates used in our previous report (Alavanja et al. 2004) included age, sex, pack-years of smoking separately for current and former smokers, and total lifetime days of pesticide use. We further evaluated potential confounding from cigarette smoking by including pack-years of cigarette smoking as a continuous variable; these two approaches yielded comparable risk estimates. We also assessed the potential for confounding by other covariates [education, body mass index, family history of lung cancer, race, state of residence, fruit and vegetable intake, alcohol consumption, and raising poultry and livestock, which is associated with reduced lung cancer incidence among farmers in the AHS (Beane Freeman et al. 2012)]; none of these variables meaningfully influenced the estimates of relative risk. In addition to adjusting for total lifetime days of pesticide application, we also conducted additional analyses adjusting for lifetime days of diazinon, dieldrin, and pendimethalin use because these pesticides were previously associated with lung cancer incidence in the AHS. Our final models included the *a priori* covariates only.

We used PROC MIANALYZE (SAS 9.3; SAS Institute Inc.) to account for our multiple imputation approach. For the pesticides dieldrin, 2,4,5-TP, parathion, chlordane, DDT, heptachlor, and toxaphene, there was no variability between the five imputed sets because their registrations had been canceled before the Phase 2 interviews were conducted. Therefore, standard proportional hazards models were used. *p*-Values for trend were calculated using natural log–transformed versions of the continuous exposure variables while adjusting for the covariates. We performed analyses stratified by smoking status to assess potential effect measure modification. In addition, we conducted analyses by lung cancer histologic type (adenocarcinoma vs. non-adenocarcinoma). These analyses are presented in Tables S1 and S2 only because the small number of lung cancer cases among strata limited precision and interpretation.

## Results

Since our previous report (Alavanja et al. 2004), 414 additional first primary, histologically confirmed incident lung cancer cases have occurred. In total, 654 first primary incident lung cancer cases were included in the present report, with an average follow-up of 14.8 years since AHS enrollment. Selected characteristics are presented in Table 1. As expected, a higher proportion of lung cancer cases than of noncases was observed with older age and greater pack-years of cigarette smoking. The proportion of lung cancer cases was slightly higher among non-whites, among those residing in North Carolina, and among those having a history of chronic lung disease. We did not find differences with sex or family history of lung cancer. Lung cancer cases were less likely to regularly consume fruits, vegetables, and alcohol than were noncases.

**Table 1:** Selected baseline characteristics of lung cancer cases and noncases, Agricultural Health Study (1993–1997).

Characteristic^*a*^	Lung cancer cases *n* = 546 (%)	Cohort members (noncases) *n* = 49,266 (%)
Age
< 55	170 (31.1)	36,434 (74.0)
55–59	114 (20.9)	4,693 (9.5)
60–64	108 (19.8)	3,754 (7.6)
65–69	78 (14.3)	2,465 (5.0)
70–74	57 (10.4)	1,307 (2.7)
≥ 75	19 (3.5)	613 (1.2)
Smoking status (pack-years)^*b*^
Never smoker	57 (10.4)	26,803 (54.4)
Former < 3.75	15 (2.8)	4,552 (9.2)
Former 3.75–15	27 (5.0)	4,128 (8.4)
Former > 15	176 (32.2)	5,405 (11.0)
Current < 11.25	26 (4.8)	1,522 (3.1)
Current 11.25–28.5	49 (9.0)	2,623 (5.3)
Current > 28.5	196 (35.9)	4,233 (8.6)
Sex
Male	535 (98.0)	48,005 (97.4)
Female	11 (2.0)	1,261 (2.6)
Race^*b*^
White	519 (95.1)	48,060 (97.8)
Black/other	27 (4.9)	1,103 (2.2)
State of residence
Iowa	231 (42.3)	32,895 (66.8)
North Carolina	315 (57.7)	16,371 (33.2)
Education (years)^*b*^
< 12	128 (24.1)	4,124 (8.5)
12	268 (50.5)	22,797 (47.2)
> 12	135 (25.4)	21,363 (44.2)
Other chronic lung disease (bronchitis and emphysema)^*b*^
No	455 (89.7)	45,165 (96.4)
Yes	52 (10.3)	1,683 (3.6)
Family history of lung cancer^*b*^
No	442 (90.4)	43,549 (93.7)
Yes	47 (9.6)	2,927 (6.3)
Vegetable intake (servings/week)^*b*^
≤ 4	173 (35.2)	15,228 (32.8)
5–7	188 (38.3)	16,913 (36.5)
> 7	130 (26.5)	14,223 (30.7)
Fruit intake (servings/week)^*b*^
≤ 2	204 (40.0)	15,313 (32.5)
3–6	189 (37.1)	18,627 (39.6)
≥ 7	117 (22.9)	13,128 (27.9)
Alcohol intake (servings/time period)^*b*^
Never	227 (44.3)	14,843 (31.4)
≤ 3/month	121 (23.6)	12,928 (27.4)
≥ 4/week	165 (32.2)	19,439 (41.2)
^***a***^Using response categories from the Agricultural Health Study enrollment questionnaire. ^***b***^Numbers do not sum to total because of missing data.		

Table 2 presents the HRs for lifetime days of use and intensity-weighted lifetime days for 13 pesticides and lung cancer. Results were included if they had been previously associated with lung cancer in the AHS [dicamba, pendimethalin, carbofuran, chlorpyrifos, and dieldrin (Alavanja et al. 2004)], in other epidemiologic studies [malathion (Pesatori et al. 1994), parathion (Pesatori et al. 1994), carbaryl (Pesatori et al. 1994), chlordane (MacMahon et al. 1988), DDT (Austin et al. 1989), and heptachlor (MacMahon et al. 1988)], or otherwise showed an association with lung cancer in this evaluation (chlorimuron ethyl). Table S3 depicts the hazard ratios for the remaining 30 pesticides, none of which was positively associated with lung cancer. Lifetime days of chlorimuron ethyl were associated with statistically significant increased risk in the highest exposure category only (HR = 1.74; 95% CI: 1.02, 2.96) but did not show an exposure–response trend (*p*_trend_ = 0.180). The highest quartile of lifetime days of pendimethalin use also showed a positive association with lung cancer (HR = 1.50; 95% CI: 0.98, 2.31). We further divided the 4th quartile at its median of lifetime days of pendimethalin. The HR for the lower 50% of the 4th quartile was 1.26 (95% CI: 0.65, 2.46), and the HR for those in the upper 50% of the 4th quartile was 2.52 (95% CI: 1.31, 4.83), although the *p* for trend was not significant (*p*_trend_ = 0.283). Lifetime days of dieldrin use also showed a positive association in the highest exposure tertile (HR = 1.93; 95% CI: 0.70, 5.30), as did the HR for the intensity-weighted lifetime exposure-days metric (HR = 2.06; 95% CI: 0.95, 4.43). The lowest and highest quartiles of lifetime days of DDT use showed a slight excess in risk, although a monotonic exposure–response gradient was not evident (*p*_trend_ = 0.695). Similarly, the highest quartile of lifetime days of malathion use showed a slight excess risk (HR = 1.35; 95% CI: 0.93, 1.97). Although parathion was only slightly associated with the risk of lung cancer in the highest quartile [(HR = 1.17; 95% CI: 0.51, 2.68) for lifetime days and (HR = 1.20; 95% CI: 0.58, 2.47) for intensity-weighted lifetime days], the test for trend was statistically significant for intensity-weighted lifetime days (*p* = 0.049) and borderline for lifetime days (*p* = 0.073). The lowest exposure category of lifetime days use for maneb/mancozeb had a statistically significant increased risk of lung cancer (HR = 3.27; 95% CI: 1.54, 2.20), but the highest exposure category was not elevated, and there was no evidence of an exposure–response gradient (*p*_trend_ = 0.939), nor were any of the other exposure categories significantly increased. Carbaryl, carbofuran, chlordane, chlorpyrifos, and heptachlor were not associated with the incidence of lung cancer. Dicamba showed a statistically significant inverse exposure–response trend, although the lowest risks were seen in the lower quartiles of exposure. Generally, the HRs for the intensity-weighed lifetime days for these pesticides were similar to the lifetime-days metric (Table 2).

**Table 2:** Hazard ratios and 95% confidence intervals for lung cancer by lifetime days pesticide exposure and intensity-weighted lifetime days, Agricultural Health Study.

Pesticide	Lifetime days			Intensity-weighted lifetime days		
	Cases (*n*)	Hazard ratio^*a*^ (95% CI)	*p* for trend	Cases (*n*)	Hazard ratio^*a*^ (95% CI)	*p* for trend
Chlorimuron ethyl (herbicide; pyrimidinylsulfonylurea)^*b*^
Nonexposed	180	1.0 (Reference)		180	1.0 (Reference)
Q1	14	1.10 (0.64, 1.90)		21	1.09 (0.69, 1.72)
Q2	37	0.96 (0.67, 1.38)		21	0.97 (0.62, 1.51)
Q3	11	1.17 (0.64, 2.16)		20	1.04 (0.65, 1.68)
Q4	16	1.74 (1.02, 2.96)	0.180	16	1.69 (1.00, 2.83)	0.294
Dicamba (herbicide; benzoic acid)
Nonexposed	293	1.0 (Reference)		293	1.0 (Reference)
Q1	38	0.64 (0.44, 0.92)		39	0.57 (0.40, 0.82)
Q2	45	0.57 (0.40, 0.83)		44	0.66 (0.47, 0.95)
Q3	45	0.75 (0.55, 1.04)		36	0.73 (0.48, 1.10)
Q4	36	0.86 (0.60, 1.24)	0.007	44	0.81 (0.59, 1.13)	0.001
Pendimethalin (herbicide; dinitroaniline)^*b*^
Nonexposed	160	1.0 (Reference)		160	1.0 (Reference)
Q1	21	1.00 (0.61, 1.62)		25	0.81 (0.52, 1.26)
Q2	33	0.85 (0.58, 1.24)		32	0.81 (0.50, 1.31)
Q3	29	0.91 (0.58, 1.42)		27	1.26 (0.82, 1.92)
Q4	28	1.50 (0.98, 2.31)	0.283	26	1.47 (0.93, 2.31)	0.551
Carbaryl (insecticide; carbamate)^*b*^
Nonexposed	112	1.0 (Reference)		112	1.0 (Reference)
Q1	58	0.93 (0.66, 1.30)		47	0.94 (0.65, 1.36)
Q2	38	0.99 (0.66, 1.49)		35	0.99 (0.67, 1.46)
Q3	33	1.15 (0.76, 1.74)		41	1.16 (0.79, 1.40)
Q4	28	1.17 (0.76, 1.79)	0.436	34	1.04 (0.70, 1.54)	0.757
Carbofuran (insecticide; chlorinated organic)
Nonexposed	336	1.0 (Reference)		336	1.0 (Reference)
Q1	40	0.76 (0.55, 1.05)		32	0.81 (0.56, 1.16)
Q2	29	0.80 (0.54, 1.19)		31	0.80 (0.55, 1.16)
Q3	23	1.08 (0.62, 1.89)		29	0.87 (0.59, 1.29)
Q4	28	0.99 (0.67, 1.47)	0.299	28	0.88 (0.59, 1.30)	0.133
Chlordane (insecticide; chlorinated organic)^*b*^
Nonexposed	169	1.0 (Reference)		169	1.0 (Reference)
Q1	22	1.57 (1.01, 2.46)		17	1.64 (0.99, 2.70)
Q2	26	1.13 (0.75, 1.71)		17	1.34 (0.81, 2.21)
Q3	12	0.95 (0.53, 1.70)		21	0.88 (0.56, 1.39)
Q4	13	1.13 (0.64, 2.01)	0.426	18	1.27 (0.78, 2.08)	0.403
Chlorpyrifos (insecticide; phosphorothioate)
Nonexposed	339	1.0 (Reference)		339	1.0 (Reference)
Q1	54	0.84 (0.63, 1.13)		44	0.91 (0.66, 1.25)
Q2	52	1.08 (0.79, 1.48)		41	0.74 (0.53, 1.03)
Q3	41	0.86 (0.61, 1.21)		40	1.03 (0.74, 1.44)
Q4	46	0.98 (0.71, 1.35)	0.497	38	0.88 (0.62, 1.25)	0.210
DDT (insecticide; chlorinated organic)^*b*^
Nonexposed	140	1.0 (Reference)		140	1.0 (Reference)
Q1	20	1.45 (0.92, 2.38)		29	1.01 (0.68, 1.52)
Q2	42	0.86 (0.61, 1.22)		27	0.96 (0.63, 1.45)
Q3	22	1.09 (0.69, 1.72)		25	0.99 (0.64, 1.53)
Q4	23	1.33 (0.84, 2.10)	0.695	26	1.46 (0.95, 2.25)	0.506
Dieldrin (insecticide; chlorinated organic)^*b*^
Nonexposed	230	1.0 (Reference)		230	1.0 (Reference)
T1	6	0.58 (0.26, 1.31)		5	1.01 (0.42, 2.47)
T2	6	1.49 (0.66, 3.37)		4	0.50 (0.18, 1.34)
T3	4	1.93 (0.70, 5.30)	0.472	7	2.06 (0.95, 4.43)	0.880
Heptachlor (insecticide; chlorinated organic)^*b*^
Nonexposed	216	1.0 (Reference)		216	1.0 (Reference)
Q1	6	1.19 (0.53, 2.68)		7	1.13 (0.53, 2.39)
Q2	11	0.65 (0.35, 1.19)		6	0.56 (0.25, 1.26)
Q3	10	0.89 (0.47, 1.68)		10	0.77 (0.41, 1.46)
Q4	5	0.66 (0.27, 1.62)	0.228	9	0.82 (0.42, 1.60)	0.193
Parathion (insecticide; phosphorothioate)^*b*^
Nonexposed	211	1.0 (Reference)		211	1.0 (Reference)
Q1	5	1.60 (0.66, 3.89)		11	1.58 (0.86, 2.91)
Q2	17	1.48 (0.90, 2.43)		9	1.37 (0.70, 2.69)
Q3	7	1.65 (0.78, 3.52)		7	1.82 (0.86, 3.89)
Q4	6	1.17 (0.51, 2.68)	0.073	8	1.20 (0.58, 2.47)	0.049
Malathion (insecticide; phosphorothioate)^*b*^
Nonexposed	78	1.0 (Reference)		78	1.0 (Reference)
Q1	28	0.98 (0.54, 1.78)		40	0.99 (0.61, 1.61)
Q2	76	1.11 (0.80, 1.52)		57	1.02 (0.72, 1.43)
Q3	35	1.00 (0.67, 1.50)		44	1.18 (0.81, 1.72)
Q4	45	1.35 (0.93, 1.97)	0.168	43	1.37 (0.94, 2.00)	0.197
Maneb/mancozeb (fungicide; dithiocarbamate)^*b*^
Nonexposed	214	1.0 (Reference)		214	1.0 (Reference)
Q1	7	3.27 (1.54, 6.97)		11	3.21 (1.74, 5.91)
Q2	11	1.39 (0.76, 2.57)		6	0.91 (0.40, 2.06)
Q3	10	1.34 (0.71, 2.53)		9	1.44 (0.74, 2.81)
Q4	5	0.72 (0.30, 1.76)	0.939	7	0.86 (0.40, 1.83)	0.436
Notes: CI, confidence interval; DDT, dichlorodiphenyltrichloroethane; Q, quartile; T, Tertile. ^***a***^Adjusted for age, smoking status and pack-years, sex, and total lifetime pesticide use. ^***b***^Lifetime-days of use were obtained from the take-home questionnaire.						

Table 3 shows the results of lagging lifetime days of exposure 5 and 15 years. The HRs from lagging lifetime exposure-days by 5 and 15 years were somewhat lower than those from unlagged analyses for pendimethalin and chlorimuron ethyl. The association between dieldrin and lung cancer incidence was not influenced because dieldrin use had ceased before either of these lag periods. No obvious pattern emerged from the lagged analysis of parathion.

**Table 3:** Hazard ratios and 95% confidence intervals for lung cancer by 5- and 15-year lagged lifetime-days pesticide exposure, Agricultural Health Study.

Pesticide	5-year lag			15-year lag		
	Cases (*n*)	Hazard ratio^*a*^ (95% CI)	*p* for trend	Cases (*n*)	Hazard ratio^*a*^ (95% CI)	*p* for trend
Chlorimuron ethyl (herbicide; pyrimidinylsulfonylurea)^*b*^
Nonexposed	181	1.0 (Reference)		206	1.0 (Reference)
Q1	16	1.24 (0.75, 2.06)		16	0.87 (0.52, 1.44)
Q2	35	0.90 (0.62, 1.31)		15	0.46 (0.27, 0.78)
Q3	10	1.15 (0.60, 2.20)		5	0.65 (0.27, 1.59)
Q4	16	1.61 (0.96, 2.71)	0.295	13	1.36 (0.77, 2.40)	0.222
Dicamba (herbicide; benzoic acid)
Nonexposed	299	1.0 (Reference)		329	1.0 (Reference)
Q1	38	0.62 (0.44, 0.88)		35	0.52 (0.37, 0.74)
Q2	43	0.54 (0.38, 0.77)		34	0.47 (0.33, 0.67)
Q3	45	0.73 (0.53, 1.00)		37	0.61 (0.43, 0.86)
Q4	33	0.79 (0.55, 1.14)	0.001	21	0.59 (0.38, 0.93)	< 0.001
Pendimethalin (herbicide; dinitroaniline)^*b*^
Nonexposed	161	1.0 (Reference)		201	1.0 (Reference)
Q1	24	1.18 (0.76, 1.85)		12	0.49 (0.26, 0.90)
Q2	30	0.78 (0.52, 1.18)		13	0.39 (0.22, 0.69)
Q3	26	0.88 (0.55, 1.41)		8	0.33 (0.16, 0.68)
Q4	25	1.31 (0.84, 2.05)	0.602	19	1.11 (0.68, 1.82)	0.003
Carbaryl (insecticide; carbamate)^*b*^
Nonexposed	112	1.0 (Reference)		131	1.0 (Reference)
Q1	55	0.87 (0.51, 1.22)		36	0.66 (0.46, 0.95)
Q2	35	0.92 (0.61, 1.34)		31	1.00 (0.67, 1.48)
Q3	29	1.20 (0.79, 1.82)		32	1.29 (0.87, 1.90)
Q4	30	1.05 (0.70, 1.59)	0.787	16	0.61 (0.36, 1.04)	0.399
Carbofuran (insecticide; carbamate)
Nonexposed	336	1.0 (Reference)		354	1.0 (Reference)
Q1	40	0.76 (0.54, 1.05)		36	0.67 (0.47, 0.94)
Q2	29	0.81 (0.55, 1.20)		23	0.67 (0.44, 1.02)
Q3	24	1.11 (0.64, 1.91)		25	1.38 (0.78, 2.43)
Q4	27	0.95 (0.64, 1.43)	0.261	18	0.63 (0.39, 1.02)	0.006
Chlordane (insecticide; chlorinated organic)^*b*^
Nonexposed	169	1.0 (Reference)		172	1.0 (Reference)
Q1	0	— (—)		1	8.72 (1.19, 64.22)
Q2	48	1.30 (0.94, 1.79)		45	1.21 (0.87, 1.69)
Q3	12	0.95 (0.53, 1.70)		11	0.89 (0.48, 1.63)
Q4	13	1.13 (0.64, 2.01)	0.424	13	1.13 (0.64, 2.00)	0.605
Chlorpyrifos (insecticide; phosphorothioate)
Nonexposed	344	1.0 (Reference)		401	1.0 (Reference)
Q1	55	0.85 (0.63, 1.13)		44	0.63 (0.46, 0.86)
Q2	49	0.98 (0.71, 1.35)		39	0.77 (0.56, 1.07)
Q3	43	0.91 (0.66, 1.26)		20	0.43 (0.28, 0.68)
Q4	41	0.86 (0.61, 1.20)	0.188	27	0.57 (0.38, 0.85)	< 0.001
DDT (insecticide; chlorinated organic)^*b*^
Nonexposed	140	1.0 (Reference)		140	1.0 (Reference)
Q1	20	1.48 (0.92, 2.38)		20	1.44 (0.90, 2.31)
Q2	42	0.86 (0.61, 1.22)		42	0.87 (0.61, 1.23)
Q3	22	1.09 (0.69, 1.72)		22	1.09 (0.69, 1.71)
Q4	23	1.33 (0.84, 2.10)	0.695	23	1.35 (0.85, 2.13)	0.709
Dieldrin (insecticide; chlorinated organic)^*b*^
Nonexposed	230	1.0 (Reference)		230	1.0 (Reference)
T1	6	0.58 (0.26, 1.31)		6	0.59 (0.26, 1.32)
T2	6	1.49 (0.66, 3.37)		6	1.44 (0.64, 3.26)
T3	4	1.93 (0.70, 5.30)	0.471	4	2.09 (0.76, 5.75)	0.468
Heptachlor (insecticide; chlorinated organic)^*b*^
Nonexposed	216	1.0 (Reference)		216	1.0 (Reference)
Q1	6	1.19 (0.53, 2.68)		6	1.16 (0.51, 2.61)
Q2	11	0.65 (0.35, 1.19)		11	0.65 (0.36, 1.20)
Q3	10	0.89 (0.47, 1.68)		10	0.90 (0.47, 1.69)
Q4	5	0.66 (0.27, 1.62)	0.228	5	0.67 (0.28, 1.64)	0.239
Parathion (insecticide; phosphorothioate)^*b*^
Nonexposed	212	1.0 (Reference)		214	1.0 (Reference)
Q1	4	1.22 (0.54, 3.28)		4	1.09 (0.40, 2.94)
Q2	17	1.49 (0.90, 2.44)		15	1.44 (0.85, 2.43)
Q3	7	1.63 (0.76, 3.47)		9	1.96 (1.00, 3.82)
Q4	6	1.17 (0.51, 2.69)	0.083	4	0.81 (0.30, 2.24)	0.168
Malathion (insecticide; phosphorothioate)^*b*^
Nonexposed	82	1.0 (Reference)		110	1.0 (Reference)
Q1	26	0.93 (0.53, 1.62)		15	0.59 (0.34, 1.02)
Q2	71	1.01 (0.73, 1.39)		53	0.69 (0.50, 0.96)
Q3	35	0.67 (0.65, 1.45)		35	0.87 (0.59, 1.28)
Q4	44	1.29 (0.89, 1.88)	0.303	32	0.85 (0.57, 1.28)	0.378
Maneb/mancozeb (fungicide; dithiocarbamate)^*b*^
Nonexposed	216	1.0 (Reference)		221	1.0 (Reference)
Q1	6	2.88 (1.27, 6.54)		6	3.20 (1.41, 7.20)
Q2	9	1.14 (0.58, 2.23)		6	0.81 (0.36, 1.83)
Q3	11	1.45 (0.79, 2.66)		10	1.40 (0.74, 2.64)	
Q4	5	0.71 (0.29, 1.74)	0.993	4	0.58 (0.22, 1.58)	0.566
Notes: CI, confidence interval; DDT, dichlorodiphenyltrichloroethane; Q, quartile; T, tertile. ^***a***^Adjusted for age, smoking status and pack-years, sex, and total lifetime pesticide use. ^***b***^Lifetime-days of use were obtained from the take-home questionnaire.						

## Discussion

With an additional 10 years of follow-up and 414 additional first primary, histologically confirmed incident lung cancer cases, we reevaluated the associations between lifetime days and intensity-weighted lifetime days for 43 pesticides and relative risk for lung cancer. Independent AHS pesticide-specific analyses for diazinon (Jones et al. 2015) and metolachlor (Silver et al. 2015) were not included here because these results have been published elsewhere. We found evidence of positive, albeit imprecise, associations with lung cancer for pendimethalin and dieldrin. These two pesticides had elevated HRs in the highest exposure category, but the exposure–response gradients were neither monotonic nor statistically significant. Parathion showed some evidence of increased risk for lung cancer, but the trends were not monotonic, nor were the excesses the largest in the highest quartile of exposure. We observed an increased hazard ratio with the use of chlorimuron ethyl in the highest exposure category. Chlorimuron ethyl use was not associated with lung cancer in a previous AHS analysis (Alavanja et al. 2004). None of the other pesticides (chlorpyrifos, carbofuran, or dicamba) was associated with lung cancer risk in this reevaluation.

Pendimethalin has been shown to induce thyroid follicular cell adenomas in rats and is classified as a possible human carcinogen (Group C) by the U.S. Environmental Protection Agency (EPA) (1997). Previous analyses of pendimethalin in the AHS (Alavanja et al. 2004; Hou et al. 2006), however, have been inconsistent. There is limited experimental evidence linking pendimethalin to genotoxicity (Dimitrov et al. 2006) or carcinogenicity in rodents (Weed Society of America 2002). To our knowledge, no epidemiologic studies other than the AHS have investigated pendimethalin use and lung cancer risk. We see only weak evidence for an association from a borderline statistically significant association with lifetime days of use and intensity-weighted lifetime days. The lung cancer excess with pendimethalin use was largely limited to the upper half of the upper quartile, but the exposure–response trends were not statistically significant.

Dieldrin is an organochlorine insecticide that was banned from agricultural use in 1970 by the U.S. EPA, although its use as a termiticide was permitted by the U.S. EPA between 1972 and 1987 (Stern 2014). There are concerns about ongoing low-level exposure because dieldrin is commonly found in hazardous waste sites and is relatively resistant to environmental degradation (Stern 2014). As with the previous analyses of the AHS cohort (Alavanja et al. 2004; Purdue et al. 2007), dieldrin was positively associated with lung cancer, but mainly in the highest tertile of use. Dieldrin has been shown to induce liver tumors in mice, but not in other rodents [International Association for Research on Cancer (IARC) 1987]. The small number of dieldrin-exposed lung cancer cases complicates interpretation here.

Parathion was recently designated by IARC as possibly carcinogenic to humans (group 2B), largely on the basis of experimental evidence (Guyton et al. 2015). To our knowledge, no previous epidemiologic studies (Pesatori et al. 1994), including our previous report (Alavanja et al. 2004), have found associations for parathion use with lung cancer specifically, although melanoma was associated with parathion use in the AHS (Dennis et al. 2010). In chronic feeding studies, parathion has been shown to be carcinogenic to Osborne-Mendel rats and to increase the incidence of alveolar/bronchiolar adenomas in B6C3F1 mice (Gulf South Research Institute et al. 1979). Furthermore, parathion has been demonstrated to damage DNA in human peripheral lymphocytes (Undeğer and Başaran 2005). In our study, the small number of exposed cases and the lack of a monotonic exposure–response gradient complicated interpretation. Although these data do not provide strong evidence to support an association, nearly all the exposure categories had excess risk and are deserving of continued investigation for a potential association between parathion and lung cancer. Malathion (Guyton et al. 2015) and DDT (Loomis et al. 2015) were also evaluated and were classified as probably carcinogenic to humans (group 2A), largely based on sufficient evidence in animals. The evidence in humans, however, was deemed limited, and the lung was not a site observed to be associated with either malathion or DDT use in the epidemiologic studies assessed. Further epidemiologic investigation of both malathion and DDT are warranted.

This is the first report from the AHS in which chlorimuron ethyl and maneb/mancozeb have been associated with lung cancer incidence. However, these new findings may be chance occurrences because they are based on relatively small numbers of exposed cases. Chlorimuron ethyl is a herbicide that was introduced in 1986 for use on soybeans. It was previously associated with wheeze among commercial applicators in the AHS (Hoppin et al. 2006). The U.S. EPA classifies chlorimuron ethyl as “not likely to be carcinogenic to humans” (U.S. EPA 2016). To our knowledge, there are no published epidemiologic reports on the relationship between chlorimuron-ethyl exposure and cancer. Maneb/mancozeb has been observed to potentiate cancer in rodents (Belpoggi et al. 2002) and to be genotoxic in cultured human lymphocytes (Srivastava et al. 2012). The U.S. EPA classifies these fungicides as probable human carcinogens (group B2) (U.S. EPA 2016). However, in the present analysis, maneb/mancozeb use was associated with lung cancer only in the lowest exposure category and did not display an exposure–response gradient.

To our knowledge, no epidemiologic studies outside of the AHS have investigated dicamba and lung cancer risk. In contrast to previous AHS evaluations, we saw no evidence of an association between dicamba and lung cancer in the present analysis with larger numbers, although *in vitro* evidence suggests that dicamba may be genotoxic (González et al. 2006, 2007). Contrary to earlier AHS evaluations, we also saw no evidence of an association between lung cancer and chlorpyrifos (Alavanja et al. 2004; Lee et al. 2004) or carbofuran (Alavanja et al. 2004; Bonner et al. 2005) use. There is experimental mechanistic evidence that chlorpyrifos can induce oxidative stress and oxidative DNA damage (Ojha and Srivastava 2014; Zafiropoulos et al. 2014) and that carbofuran may be genotoxic (Mladinic et al. 2012). The proportion of AHS cohort members using either chlorpyrifos or carbofuran has declined since enrollment (Hoppin et al. 2012). Our analysis focused on the active ingredients of formulated mixtures of commercial products. These formulations contain both active ingredients and so-called “inert ingredients,” and we cannot rule out the possibility that changes in the formulated mixtures associated with dicamba, chlorpyrifos, and carbofuran products are associated with changes in observed associations. Conversely, previous associations observed between these chemicals and lung cancer with fewer cases may have been due to chance.

We observed a number of inverse associations with lagged exposures, particularly for the 15-year exposure lag. We cannot explain these inverse associations in our data; none of these inverse associations is supported by biologic evidence, however. Rather, the limited evidence that does exist suggests carcinogenic potential as previously noted for, for example, dicamba, chlorpyrifos, and maneb/mancozeb.

Several limitations are evident in the present analysis. Despite an additional 10 years of follow-up and a substantial accrual of lung cancer cases, the number of lung cancer cases exposed to some pesticides remains small and continues to hamper study precision as well as our ability to evaluate risk by histologic type of lung cancer and to explore effect modification by smoking, particularly for chemicals for which patterns of use information were collected only with the take-home questionnaire. In addition, the analysis relies on imputed pesticide use data for a substantial fraction of the cohort.

We cannot rule out the possibility for chance or multiple comparisons to explain some of our results. Although approaches to adjust for multiple comparisons exist, a number of authors have warned against using such measures in epidemiological studies (Rothman 1990; Savitz and Olshan 1995; Goldberg and Silbergeld 2011). Our goal was to describe the magnitude of associations between specific pesticides and lung cancer risk. As such, we prefer to let other epidemiological studies and other relevant evidence (e.g., toxicological data) help sort out the likely reality of the findings.

Although the reliability of information on pesticide use obtained from farmers is quite good and is comparable to that from other factors commonly obtained by questionnaire in epidemiologic studies such as smoking and alcohol consumption (Blair et al. 2002), some exposure assessment error undoubtedly occurs. In this prospective cohort study, exposure misclassification is likely to diminish estimates of relative risk and to mute any real exposure–response relationships (Blair et al. 2011).

Although information on smoking was included in the statistical models, the possibility of residual confounding by active smoking and secondhand smoke exposure should be considered. This possibility seems unlikely, however, because there was no evidence of a link between smoking and pesticide use. Links were certainly not evident with many pesticides because the use of most pesticides did not result in an increase in the relative risk of lung cancer. Thus, any residual confounding would have to be chemical-specific. We evaluated a number of factors, including use of other pesticides (diazinon, pendimethalin, dieldrin, and chlorimuron ethyl) that might potentially confound associations between specific pesticides and lung cancer, none of which meaningfully influenced the risk estimates in our analyses. Exposure to secondhand smoke was not ascertained in the AHS; however, any confounding resulting from secondhand smoke is likely to be small in comparison to direct smoking.

There is the possibility that a healthy worker survivor effect (HWSE) may have attenuated or reversed the reported associations. Unfortunately, we cannot carefully evaluate for an HWSE because time-dependent exposure information before enrollment was not collected. Nonetheless, the likelihood of an HWSE is low in the AHS cohort because the participants are predominately farm owners/operators who have a sizable economic investment in their operation, providing an incentive to continue farming.

This study has a number of strengths. The study population comprises a large population of farmers and commercial pesticide applicators who can provide detailed and reliable information regarding their pesticide use history (Blair et al. 2002). Information on pesticide use, application practices, and other information was obtained before the onset of cancer, diminishing the chances of case–response bias. Loss to follow-up is minimal owing to the use of high-quality state cancer registries and vital records and to the low residential mobility of this cohort. An algorithm that incorporated several exposure determinants that predicted urinary pesticide levels was used to develop an intensity-weighted exposure metric in our study (Coble et al. 2011). Information on potential confounders, such as smoking and the use of other pesticides, was available and could be evaluated and controlled in the analysis.

## Conclusion

Several epidemiologic studies have found associations between pesticides and lung cancer (Alavanja and Bonner 2012). In our continuing survey within the AHS, we have found that no specific class of pesticide is associated with lung cancer. Although the results were not entirely consistent, we did observe some evidence of associations with pendimethalin and dieldrin. In addition, we found possible new associations for chlorimuron ethyl and parathion with lung cancer that have not been previously observed in the AHS and deserve further evaluation.

## Supplemental Material

(105 KB) PDF

## References

[r1] Alavanja MC, Bonner MR (2012). Occupational pesticide exposures and cancer risk: a review.. J Toxicol Environ Health B Crit Rev.

[r2] Alavanja MC, Dosemeci M, Samanic C, Lubin J, Lynch CF, Knott C (2004). Pesticides and lung cancer risk in the Agricultural Health Study cohort.. Am J Epidemiol.

[r3] Alavanja MC, Sandler DP, McMaster SB, Zahm SH, McDonnell CJ, Lynch CF (1996). The Agricultural Health Study.. Environ Health Perspect.

[r4] American Cancer Society (2017). *Cancer Facts & Figures 2017*.. https://www.cancer.org/content/dam/cancer-org/research/cancer-facts-and-statistics/annual-cancer-facts-and-figures/2017/cancer-facts-and-figures-2017.pdf.

[r5] Austin H, Keil JE, Cole P (1989). A prospective follow-up study of cancer mortality in relation to serum DDT.. Am J Public Health.

[r6] Barthel E (1981). Increased risk of lung cancer in pesticide-exposed male agricultural workers.. J Toxicol Environ Health.

[r7] Beane Freeman LE, Deroos AJ, Koutros S, Blair A, Ward MH, Alavanja M (2012). Poultry and livestock exposure and cancer risk among farmers in the Agricultural Health Study.. Cancer Causes Control.

[r8] Becher H, Flesch-Janys D, Kauppinen T, Kogevinas M, Steindorf K, Manz A (1996). Cancer mortality in German male workers exposed to phenoxy herbicides and dioxins.. Cancer Causes Control.

[r9] Belpoggi F, Soffritti M, Guarino M, Lambertini L, Cevolani D, Maltoni C (2002). Results of long-term experimental studies on the carcinogenicity of ethylene-bis-dithiocarbamate (Mancozeb) in rats.. Ann N Y Acad Sci.

[r10] Blair A, Dosemeci M, Heineman EF (1993). Cancer and other causes of death among male and female farmers from twenty-three states.. Am J Ind Med.

[r11] Blair A, Freeman LB (2009). Epidemiologic studies in agricultural populations: observations and future directions.. J Agromedicine.

[r12] Blair A, Grauman DJ, Lubin JH, Fraumeni JF (1983). Lung cancer and other causes of death among licensed pesticide applicators.. J Natl Cancer Inst.

[r13] Blair A, Tarone R, Sandler D, Lynch CF, Rowland A, Wintersteen W (2002). Reliability of reporting on life-style and agricultural factors by a sample of participants in the Agricultural Health Study from Iowa.. Epidemiology.

[r14] Blair A, Thomas K, Coble J, Sandler DP, Hines CJ, Lynch CF (2011). Impact of pesticide exposure misclassification on estimates of relative risks in the Agricultural Health Study.. Occup Environ Med.

[r15] Blair A, Zahm SH, Pearce NE, Heineman EF, Fraumeni JF (1992). Clues to cancer etiology from studies of farmers.. Scand J Work Environ Health.

[r16] Bonner MR Lee WJ Sandler DP Hoppin JA Dosemeci M Alavanja MC 2005 Occupational exposure to carbofuran and the incidence of cancer in the Agricultural Health Study. Environ Health Perspect 113 285 289, doi:10.1289/ehp.7451 15743716 PMC1253753

[r17] Coble J, Thomas KW, Hines CJ, Hoppin JA, Dosemeci M, Curwin B (2011). An updated algorithm for estimation of pesticide exposure intensity in the Agricultural Health Study.. Int J Environ Res Public Health.

[r18] Dennis LK Lynch CF Sandler DP Alavanja MC 2010 Pesticide use and cutaneous melanoma in pesticide applicators in the Agricultural Heath Study. Environ Health Perspect 118 812 817, doi:10.1289/ehp.0901518 20164001 PMC2898858

[r19] Dimitrov BD, Gadeva PG, Benova DK, Bineva MV (2006). Comparative genotoxicity of the herbicides Roundup, Stomp and Reglone in plant and mammalian test systems.. Mutagenesis.

[r20] Dosemeci M, Alavanja MC, Rowland AS, Mage D, Zahm SH, Rothman N (2002). A quantitative approach for estimating exposure to pesticides in the Agricultural Health Study.. Ann Occup Hyg.

[r21] Goldberg M, Silbergeld E (2011). On multiple comparisons and on the design and interpretation of epidemiological studies of many associations.. Environ Res.

[r22] González NV, Soloneski S, Larramendy ML (2006). Genotoxicity analysis of the phenoxy herbicide dicamba in mammalian cells *in vitro*.. Toxicology in Vitro.

[r23] González NV, Soloneski S, Larramendy ML (2007). The chlorophenoxy herbicide dicamba and its commercial formulation banvel induce genotoxicity and cytotoxicity in Chinese hamster ovary (CHO) cells.. Mutat Res.

[r24] Gulf South Research Institute; Carcinogenesis Testing Program; National Institutes of Health (1979). *Bioassay of Parathion for Possible Carcinogenicity*. National Cancer Institute carcinogenesis technical report series 70..

[r25] Guyton KZ, Loomis D, Grosse Y, El Ghissassi F, Benbrahim-Tallaa L, Guha N (2015). Carcinogenicity of tetrachlorvinphos, parathion, malathion, diazinon, and glyphosate.. Lancet Oncol.

[r26] Heltshe SL, Lubin JH, Koutros S, Coble JB, Ji BT, Alavanja MC (2012). Using multiple imputation to assign pesticide use for non-responders in the follow-up questionnaire in the Agricultural Health Study.. J Expo Sci Environ Epidemiol.

[r27] Hines CJ, Deddens JA, Jaycox LB, Andrews RN, Striley CA, Alavanja MC (2008). Captan exposure and evaluation of a pesticide exposure algorithm among orchard pesticide applicators in the Agricultural Health Study.. Ann Occup Hyg.

[r28] Hoppin JA, Long S, Umbach DM, Lubin JH, Starks SE, Gerr F (2012). Lifetime organophosphorous insecticide use among private pesticide applicators in the Agricultural Health Study.. J Expo Sci Environ Epidemiol.

[r29] Hoppin JA, Umbach DM, London SJ, Lynch CF, Alavanja MC, Sandler DP (2006). Pesticides associated with wheeze among commercial pesticide applicators in the Agricultural Health Study.. Am J Epidemiol.

[r30] Hou L, Lee WJ, Rusiecki J, Hoppin JA, Blair A, Bonner MR (2006). Pendimethalin exposure and cancer incidence among pesticide applicators.. Epidemiology.

[r31] IARC (International Agency for Research on Cancer) (1987). Overall evaluation of carcinogenicity: an updating of IARC Monographs volumes 1 to 42.. IARC Mongr Eval Carcinog Risk Hum suppl 7.

[r32] Jones RR, Barone-Adesi F, Koutros S, Lerro CC, Blair A, Lubin J (2015). Incidence of solid tumours among pesticide applicators exposed to the organophosphate insecticide diazinon in the Agricultural Health Study: an updated analysis.. Occup Environ Med.

[r33] Lee WJ, Blair A, Hoppin JA, Lubin JH, Rusiecki JA, Sandler DP (2004). Cancer incidence among pesticide applicators exposed to chlorpyrifos in the Agricultural Health Study.. J Natl Cancer Inst.

[r34] Loomis D, Guyton K, Grosse Y, El Ghissasi F, Bouvard V, Benbrahim-Tallaa L (2015). Carcinogenicity of lindane, DDT, and 2,4-dichlorophenoxyacetic acid.. Lancet Oncol.

[r35] MacMahon B, Monson RR, Wang HH, Zheng TZ (1988). A second follow-up of mortality in a cohort of pesticide applicators.. J Occup Med.

[r36] Maroni M, Colosio C, Ferioli A, Fait A (2000). Biological monitoring of pesticide exposure: a review. Introduction.. Toxicology.

[r37] Mladinic M, Zeljezic D, Shaposhnikov SA, Collins AR (2012). The use of FISH-comet to detect *c-Myc* and *TP 53* damage in extended-term lymphocyte cultures treated with terbuthylazine and carbofuran.. Toxicol Lett.

[r38] Ojha A, Srivastava N (2014). *In vitro* studies on organophosphate pesticides induced oxidative DNA damage in rat lymphocytes.. Mutat Res Genet Toxicol Environ Mutagen.

[r39] Pesatori AC, Sontag JM, Lubin JH, Consonni D, Blair A (1994). Cohort mortality and nested case-control study of lung cancer among structural pest control workers in Florida (United States).. Cancer Causes Control.

[r40] Purdue MP, Hoppin JA, Blair A, Dosemeci M, Alavanja MC (2007). Occupational exposure to organochlorine insecticides and cancer incidence in the Agricultural Health Study.. Int J Cancer.

[r41] Rothman KJ (1990). No adjustments are needed for multiple comparisons.. Epidemiology.

[r42] Savitz DA, Olshan AF (1995). Multiple comparisons and related issues in the interpretation of epidemiologic data.. Am J Epidemiol.

[r43] Silver SR, Bertke SJ, Hines CJ, Alavanja MC, Hoppin JA, Lubin JH (2015). Cancer incidence and metolachlor use in the Agricultural Health Study: an update.. Int J Cancer.

[r44] Srivastava AK, Ali W, Singh R, Bhui K, Tyagi S, Al-Khedhairy AA (2012). Mancozeb-induced genotoxicity and apoptosis in cultured human lymphocytes.. Life Sci.

[r45] Stern AH (2014). Hazard identification of the potential for dieldrin carcinogenicity to humans.. Environ Res.

[r46] Thomas KW, Dosemeci M, Coble JB, Hoppin JA, Sheldon LS, Chapa G (2010). Assessment of a pesticide exposure intensity algorithm in the Agricultural Health Study.. J Expo Sci Environ Epidemiol.

[r47] Torre LA, Bray F, Siegel RL, Ferlay J, Lortet-Tieulent J, Jemal A (2015). Global cancer statistics, 2012.. CA Cancer J Clin.

[r48] U.S. EPA (Environmental Protection Agency) (1997). *R.E.D. Facts: Pendimethalin*..

[r49] U.S. EPA (2016). Chemicals Evaluated for Carcinogenic Potential. Annual Cancer Report 2016.. http://npic.orst.edu/chemicals_evaluated.pdf.

[r50] Undeğer U1, Başaran N (2005). Effects of pesticides on human peripheral lymphocytes in vitro: induction of DNA damage.. Arch Toxicol.

[r51] Weed Society of America (2002). *Herbicide Handbook of the Weed Society of America*. 8th ed..

[r52] Zafiropoulos A, Tsarouhas K, Tsitsimpikou C, Fragkiadaki P, Germanakis I, Tsardi M (2014). Cardiotoxicity in rabbits after a low-level exposure to diazinon, propoxur, and chlorpyrifos.. Hum Exp Toxicol.

