# Supplementary material for: Occupational Exposure to Pesticides and the Incidence of Lung Cancer in the Agricultural Health Study
Source: Environ Health Perspect. 2016 Jul 6;125(4):544–51. doi: 10.1289/EHP456 (PMC5381995; doi:10.1289/EHP456)
Supplement: (105 KB) PDF [file EHP456.s001.acco.pdf]

Environ Health Perspect
DOI: 10.1289/EHP456

Note to readers with disabilities: *EHP* strives to ensure that all journal content is accessible to all readers. However, some figures and Supplemental Material published in *EHP* articles may not conform to 508 standards due to the complexity of the information being presented. If you need assistance accessing journal content, please contact ehp508@niehs.nih.gov. Our staff will work with you to assess and meet your accessibility needs within 3 working days.

Supplemental Material

Occupational Exposure to Pesticides and the Incidence of Lung Cancer in the Agricultural Health Study

Matthew R. Bonner, Laura E. Beane Freeman, Jane A. Hoppin, Stella Koutros, Dale P. Sandler, Charles F. Lynch, Cynthia J. Hines, Kent Thomas, Aaron Blair, and Michael C.R. Alavanja

Table of Contents

Table S1. Lung cancer risk among applicators by lifetime exposure-days of indicated pesticide, stratified by smoking status, Agricultural Health Study

Table S2. Lung cancer risk among applicators by lifetime exposure-days of indicated pesticide, stratified by histology, Agricultural Health Study

Table S3. Hazard ratios and 95% confidence limits for lung cancer by lifetime days of pesticide exposure and intensity-weighted lifetime exposure to 30 pesticides, Agricultural Health Study

Table S1. Lung Cancer risk among applicators by lifetime exposure-days of indicated pesticide, stratified by smoking status, Agricultural Health Study

Pesticide by lifetime exposure-days	Never Smokers			Former Smokers			Current Smokers			
	Cases (*n*)	Hazard Ratio	95% confidence interval	Cases (*n*)	Hazard Ratio	95% confidence interval	Cases (*n*)	Hazard Ratio	95% confidence interval	P for Interaction
Chlorimuron-ethyl (herbicide; pyrimidinylsulfonylurea) [$]										
Non-exposed	16	1.0	Referent	84	1.0	Referent	80	1.0	Referent	
≤ median	8	2.17	0.92-5.11	10	0.58	0.30-1.12	24	1.09	0.69-1.73	
> median	7	3.80	1.53-9.48	12	1.22	0.66-2.26	17	1.10	0.59-1.73	
P_{trend}		0.001			0.987			0.872		0.107
Dicamba (herbicide; benzoic acid)										
No exposure	27	1.0	Referent	118	1.0	Referent	148	1.0	Referent	
≤ median	13	0.68	0.35-1.32	32	0.59	0.40-0.87	38	0.63	0.44-0.90	
> median	7	0.59	0.25-1.38	32	0.74	0.50-1.10	42	0.89	0.63-1.25	
P_{trend}		0.464			0.024			0.182		0.311
Pendimethalin (herbicide; dinitroaniline) [$]										
No exposure	20	1.0	Referent	70	1.0	Referent	70	1.0	Referent	
≤ median	8	1.11	0.49-2.54	18	0.74	0.44-1.25	28	0.95	0.61-1.48	
> median	2	0.41	0.10-1.80	20	1.19	0.72-1.99	35	1.27	0.82-1.96	
P_{trend}		0.585			0.678			0.206		0.141
Carbaryl (insecticide; carbamate) [$]										
No exposure	15	1.0	Referent	42	1.0	Referent	55	1.0	Referent	
≤ median	9	0.80	0.35-1.83	47	1.08	0.71-1.63	40	0.79	0.53-1.19	
> median	8	1.73	0.72-4.17	25	1.06	0.64-1.75	28	1.11	0.69-1.76	
P_{trend}		0.538			0.509			0.856		0.612
Carbofuran (insecticide; carbamate)										
No exposure	40	1.0	Referent	127	1.0	Referent	169	1.0	Referent	
≤ median	6	0.48	0.20-1.14	27	0.80	0.53-1.21	36	0.87	0.60-1.24	
> median	2	0.30	0.07-1.23	23	1.21	0.77-1.90	26	0.89	0.58-1.36	
P_{trend}		0.049			0.927			0.586		0.213

Table S1 (continued). Lung Cancer risk among applicators by lifetime exposure-days of indicated pesticide, stratified by smoking status.

Pesticide by lifetime exposure-days	Never Smokers			Former Smokers			Current Smokers			
	Cases (*n*)	Hazard Ratio	95% confidence interval	Cases (*n*)	Hazard Ratio	95% confidence interval	Cases (*n*)	Hazard Ratio	95% confidence interval	P for Interaction
Chlordane (insecticide; chlorinated organic) [$]										
No exposure	20	1.0	Referent	69	1.0	Referent	80	1.0	Referent	
≤ median	9	1.98	0.90-4.36	18	0.99	0.59-1.67	21	1.47	0.91-2.38	
> median	1	0.36	0.05-2.70	12	1.01	0.55-1.87	12	1.27	0.69-2.34	
P_{trend}		0.889			0.750			0.348		0.343
Chlorpyrifos (insecticide; phosphorothioate)										
No exposure	41	1.0	Referent	138	1.0	Referent	160	1.0	Referent	
≤ median	8	0.57	0.27-1.22	47	1.03	0.74-1.43	51	0.92	0.67-1.26	
> median	5	0.43	0.17-1.09	28	0.81	0.53-1.22	54	1.14	0.83-1.57	
P_{trend}		0.068			0.670			0.864		0.017
DDT (insecticide; chlorinated organic) [$]										
No exposure	14	1.0	Referent	55	1.0	Referent	71	1.0	Referent	
≤ median	10	1.56	0.67-3.61	28	0.87	0.55-1.37	24	1.01	0.63-1.61	
> median	6	1.85	0.68-5.02	20	1.22	0.72-2.06	19	1.06	0.62-1.80	
P_{trend}		0.336			0.881			0.965		0.453
Malathion (insecticide; phosphorothioate) [$]										
No exposure	10	1.0	Referent	27	1.0	Referent	41	1.0	Referent	
≤ median	13	0.97	0.43-2.23	51	1.42	0.89-2.27	40	0.91	0.59-1.41	
> median	11	1.24	0.52-2.94	34	1.18	0.71-1.97	35	1.17	0.74-1.86	
P_{trend}		0.551			0.596			0.299		0.940

*Hazard ratios adjusted for smoking (pack-years among current and pack-years among former smokers), age, gender, and total days of any pesticide application

[$]Lifetime-days of use were obtained from the take home questionnaire

Table S2. Lung cancer risk among applicators by lifetime exposure-days of indicated pesticide, stratified by histology, Agricultural Health Study

Pesticide by lifetime exposure-days	Adenocarcinoma			Non-Adenocarcinoma		
	Cases (*n*)	Hazard Ratio	95% confidence interval	Cases (*n*)	Hazard Ratio	95% confidence interval
Chlorimuron-ethyl (herbicide; pyrimidinylsulfonylurea) $						
No exposure	52	1.0	Referent	128	1.0	Referent
≤median	13	1.03	0.55-1.95	29	0.98	0.65-1.48
>median	6	0.77	0.33-1.82	30	1.49	0.99-2.25
P_{trend}		0.415			0.047	
Dicamba (herbicide; benzoic acid)						
No exposure	74	1.0	Referent	219	1.0	Referent
≤median	22	0.60	0.35-1.01	61	0.61	0.45-0.83
>median	24	0.96	0.63-1.48	57	0.81	0.62-1.05
P_{trend}		0.331			0.010	
Pendimethalin (herbicide; dinitroaniline) $						
No exposure	47	1.0	Referent	113	1.0	Referent
≤median	12	0.92	0.62-1.36	42	1.15	0.58-2.27
>median	12	1.45	0.92-2.30	45	0.71	0.34-1.48
P_{trend}		0.677			0.138	
Carbaryl (insecticide; carbamate) $						
No exposure	28	1.0	Referent	84	1.0	Referent
≤median	28	1.09	0.64-1.86	68	0.91	0.65-1.26
>median	14	1.05	0.55-2.04	47	1.20	0.84-1.75
P_{trend}		0.712			0.459	
Carbofuran (insecticide; carbamate)						
No exposure	91	1.0	Referent	245	1.0	Referent
≤median	15	0.62	0.36-1.08	54	0.83	0.62-1.12
>median	10	0.71	0.37-1.38	41	1.00	0.72-1.41
P_{trend}		0.168			0.680	
Chlordane (insecticide; chlorinated organic) $						
No exposure	46	1.0	Referent	123	1.0	Referent
≤median	17	1.60	0.92-2.80	31	1.19	0.80-1.76
>median	5	0.73	0.29-1.85	20	1.16	0.72-1.87
P_{trend}		0.977			0.343	
Chlorpyrifos (insecticide; phosphorothioate)						
No exposure	86	1.0	Referent	253	1.0	Referent
≤median	25	0.92	0.57-1.47	81	0.96	0.74-1.23
>median	20	0.85	0.51-1.41	67	0.94	0.71-1.24
P_{trend}		0.530			0.666	
DDT (insecticide; chlorinated organic) $						
No exposure	36	1.0	Referent	104	1.0	Referent
≤median	17	1.00	0.56-1.80	45	0.99	0.79-1.42
>median	15	1.52	0.82-2.84	30	1.11	0.73-1.69
P_{trend}		0.424			0.961	
Malathion (insecticide; phosphorothioate) $						
No exposure	22	1.0	Referent	56	1.0	Referent
≤median	32	1.07	0.60-1.90	72	1.08	0.74-1.57
>median	18	0.86	0.46-1.62	62	1.31	0.91-1.90
P_{trend}		0.528			0.041	

*Hazard ratios adjusted for smoking (pack-years among current and pack-years among former smokers), age, gender, and total days of any pesticide application.

$Lifetime-days of use were obtained from the take home questionnaire

Table S3. Hazard Ratios and 95% Confidence Limits for Lung Cancer by Lifetime Days of Pesticide Exposure and Intensity-Weighted Lifetime Exposure to 30 Pesticides, Agricultural Health Study

Lifetime Days Pesticide Exposure					Intensity-Weighted Lifetime Exposure Days				
Pesticide	Cases (n)	Hazard* Ratio	95% Confidence Interval	P for Trend		Cases (n)	Hazard* Ratio	95% Confidence Interval	P for trend
**Atrazine**									
Non-exposed	169	1.00	Ref.		Non-exposed	169	1.00	Ref.	
Q1	76	1.10	0.84-1.44		Q1	93	1.25	0.93-1.66	
Q2	119	0.95	0.73-1.24		Q2	93	0.98	0.76-1.26	
Q3	85	1.04	0.80-1.36		Q3	87	0.89	0.68-1.17	
Q4	78	0.84	0.64-1.10	0.177	Q4	85	0.86	0.65-1.15	0.187
**Cyanazine**									
Non-exposed	303	1.00	Ref.		Non-exposed	303	1.00	Ref.	
Q1	11	0.95	0.67-1.35		Q1	35	1.01	0.54-1.89	
Q2	81	0.89	0.64-1.22		Q2	42	0.87	0.68-1.11	
Q3	28	0.75	0.53-1.05		Q3	40	0.71	0.48-1.05	
Q4	35	0.87	0.61-1.22	0.144	Q4	38	0.91	0.63-1.30	0.250
**EPTC**									
Non-exposed	382	1.00	Ref.		Non-exposed	382	1.00	Ref.	
Q1	11	1.06	0.58-1.94		Q1	13	0.66	0.38-1.15	
Q2	26	0.74	0.50-1.11		Q2	17	1.00	0.61-1.62	
Q3	15	1.01	0.61-1.70		Q3	23	1.26	0.83-1.92	
Q4	13	1.08	0.62-1.90	0.602	Q4	12	0.70	0.39-1.25	0.300
**Alachlor**									
Non-exposed	223	1.00	Ref.		Non-exposed	223	1.00	Ref.	
Q1	58	1.00	0.74-1.33		Q1	66	0.99	0.75-1.31	
Q2	84	0.85	0.66-1.10		Q2	70	0.84	0.64-1.10	
Q3	59	1.11	0.83-1.48		Q3	60	1.08	0.81-1.43	
Q4	42	0.78	0.55-1.10	0.285	Q4	47	0.81	0.59-1.13	0.200
**Metribuzin**$^{\$}$									
Non-exposed	175	1.00	Ref.		Non-exposed	175	1.00	Ref.	
Q1	10	1.05	0.55-2.00		Q1	15	0.93	0.54-1.59	
Q2	39	0.80	0.57-1.14		Q2	27	0.74	0.49-1.12	
Q3	14	1.29	0.74-2.25		Q3	19	1.45	0.90-2.34	
Q4	15	1.17	0.68-2.00	0.611	Q4	17	1.07	0.64-1.78	0.373
**Paraquat**$^{\$}$									
Non-exposed	198	1.00	Ref.		Non-exposed	198	1.00	Ref.	
Q1	10	1.02	0.50-2.08		Q1	13	1.03	0.58-1.80	
Q2	22	1.23	0.78-1.91		Q2	15	1.33	0.75-2.35	
Q3	7	0.98	0.45-2.12		Q3	13	1.00	0.57-1.75	
Q4	16	1.24	0.73-2.10	0.678	Q4	14	1.35	0.77-2.36	0.537

Lifetime Days Pesticide Exposure					Intensity-Weighted Lifetime Exposure Days				
Pesticide	Cases (n)	Hazard* Ratio	95% Confidence Interval	P for Trend		Cases (n)	Hazard* Ratio	95% Confidence Interval	P for trend
**Petroleum Oil**[$]									
Non-exposed	197	1.00	Ref.		Non-exposed	197	1.00	Ref.	
Q1	4	0.79	0.29-2.14		Q1	11	0.93	0.51-1.71	
Q2	24	1.05	0.68-1.60		Q2	17	1.11	0.67-1.82	
Q3	11	1.32	0.72-2.42		Q3	12	1.20	0.67-2.15	
Q4	15	1.33	0.78-2.27	0.505	Q4	14	1.35	0.78-2.34	0.740
**Imazethapyr**									
Non-exposed	318	1.00	Ref.		Non-exposed	318	1.00	Ref.	
Q1	22	1.28	0.82-2.01		Q1	25	0.82	0.54-1.26	
Q2	37	0.73	0.52-1.03		Q2	35	0.83	0.58-1.19	
Q3	38	0.81	0.57-1.15		Q3	35	0.90	0.63-1.28	
Q4	38	0.88	0.62-1.24	0.616	Q4	39	0.85	0.60-1.19	0.268
**Glyphosate**									
Non-exposed	92	1.00	Ref.		Non-exposed	92	1.00	Ref.	
Q1	26	1.09	0.67-1.76		Q1	104	1.02	0.75-1.38	
Q2	159	1.12	0.85-1.45		Q2	120	1.21	0.91-1.61	
Q3	153	1.18	0.89-1.55		Q3	111	1.26	0.95-1.68	
Q4	101	1.15	0.85-1.55	0.693	Q4	104	1.10	0.82-1.48	0.686
**Butylate**[$]									
Non-exposed	192	1.00	Ref.		Non-exposed	192	1.00	Ref.	
Q1	13	0.59	0.33-1.03		Q1	17	0.77	0.47-1.27	
Q2	22	1.41	0.90-2.20		Q2	13	1.16	0.66-2.05	
Q3	11	1.72	0.94-3.17		Q3	12	1.52	0.84-2.72	
Q4	12	0.98	0.54-1.78	0.842	Q4	16	1.05	0.62-1.78	0.551
**Trifluralin**									
Non-exposed	247	1.00	Ref.		Non-exposed	247	1.00	Ref.	
Q1	45	0.98	0.71-1.35		Q1	46	0.86	0.62-1.19	
Q2	78	0.84	0.65-1.09		Q2	64	0.82	0.62-1.10	
Q3	40	0.80	0.56-1.15		Q3	50	0.93	0.68-1.26	
Q4	49	0.72	0.53-1.00	0.136	Q4	52	0.73	0.54-1.00	0.113
**2,4-D**									
Non-exposed	153	1.00	Ref.		Non-exposed	153	1.00	Ref.	
Q1	112	0.76	0.58-0.98		Q1	97	0.77	0.59-1.02	
Q2	97	0.75	0.58-0.97		Q2	99	0.70	0.54-0.91	
Q3	86	0.79	0.60-1.03		Q3	87	0.84	0.64-1.09	
Q4	84	0.74	0.56-0.97	0.41	Q4	95	0.75	0.57-0.98	0.195

Lifetime Days Pesticide Exposure					Intensity-Weighted Lifetime Exposure Days				
Pesticide	Cases (n)	Hazard* Ratio	95% Confidence Interval	P for Trend		Cases (n)	Hazard* Ratio	95% Confidence Interval	P for trend
**2,4,5-T**[$]									
Non-exposed	187	1.00	Ref.		Non-exposed	187	1.00	Ref.	
Q1	10	0.84	0.43-1.64		Q1	14	0.75	0.43-1.32	
Q2	21	0.71	0.45-1.12		Q2	17	0.89	0.54-1.47	
Q3	12	1.02	0.57-1.83		Q3	13	0.68	0.39-1.20	
Q4	16	1.17	0.70-1.96	0.773	Q4	15	1.47	0.86-2.51	0.939
**Permethrin** (crops)									
Non-exposed	394	1.00	Ref.		Non-exposed	394	1.00	Ref.	
Q1	11	1.18	0.64-2.16		Q1	13	1.18	0.68-2.05	
Q2	14	0.87	0.50-1.51		Q2	16	0.82	0.48-1.40	
Q3	18	0.90	0.54-1.50		Q3	14	1.03	0.60-1.78	
Q4	13	1.27	0.72-2.25	0.749	Q4	13	1.10	0.62-1.96	0.377
**Permethrin** (animals)									
Non-exposed	428	1.00	Ref.		Non-exposed	428	1.00	Ref.	
Q1	3	1.37	0.43-4.27		Q1	7	0.62	0.25-1.51	
Q2	6	0.59	0.23-1.50		Q2	9	1.02	0.48-2.14	
Q3	15	0.98	0.57-1.70		Q3	9	0.92	0.45-1.86	
Q4	6	0.65	0.27-1.59	0.718	Q4	5	0.72	0.29-1.73	0.492
**Terbufos**									
Non-exposed	316	1.00	Ref.		Non-exposed	316	1.00	Ref.	
Q1	64	0.93	0.71-1.21		Q1	40	0.89	0.64-1.24	
Q2	30	0.86	0.59-1.26		Q2	34	1.02	0.72-1.45	
Q3	23	0.88	0.57-1.36		Q3	34	0.88	0.62-1.26	
Q4	19	0.84	0.53-1.36	0.34	Q4	28	0.79	0.53-1.17	0.701
**Fonofos**									
Non-exposed	373	1.00	Ref.		Non-exposed	373	1.00	Ref.	
Q1	29	1.24	0.85-1.81		Q1	19	1.22	0.77-1.96	
Q2	17	0.83	0.51-1.36		Q2	23	0.88	0.58-1.34	
Q3	21	0.88	0.57-1.36		Q3	24	0.93	0.62-1.41	
Q4	21	1.20	0.76-1.90	0.858	Q4	22	1.23	0.80-1.90	0.669
**Lindane**[$]									
Non-exposed	213	1.00	Ref.		Non-exposed	213	1.00	Ref.	
Q1	11	0.69	0.37-1.31		Q1	8	0.60	0.29-1.28	
Q2	11	1.19	0.63-2.24		Q2	12	1.09	0.59-2.00	
Q3	10	1.20	0.61-2.35		Q3	7	0.94	0.44-2.01	
Q4	5	1.05	0.43-2.58	0.676	Q4	10	1.60	0.82-3.14	0.754

Lifetime Days Pesticide Exposure					Intensity-Weighted Lifetime Exposure Days				
**Pesticide**	**Cases (n)**	**Hazard* Ratio**	**95% Confidence Interval**	**P for Trend**		**Cases (n)**	**Hazard* Ratio**	**95% Confidence Interval**	**P for trend**
**Aldicarb**$^{\$}$									
Non-exposed	223	1.00	Ref.		Non-exposed	223	1.00	Ref.	
Q1	5	2.13	0.85-5.31		Q1	8	1.36	0.64-2.88	
Q2	14	0.99	0.51-1.91		Q2	5	1.18	0.48-2.92	
Q3	4	0.80	0.24-2.62		Q3	5	0.91	0.36-2.34	
Q4	5	0.81	0.26-2.51	0.379	Q4	3	0.72	0.21-2.50	0.504
**Phorate**$^{\$}$									
Non-exposed	182	1.00	Ref.		Non-exposed	182	1.00	Ref.	
Q1	7	0.74	0.35-1.57		Q1	13	0.69	0.39-1.21	
Q2	26	0.70	0.47-1.06		Q2	20	0.71	0.44-1.12	
Q3	16	1.05	0.63-1.75		Q3	15	1.14	0.67-1.93	
Q4	14	1.43	0.82-2.47	0.837	Q4	15	1.35	0.79-2.29	0.758
**Aldrin**$^{\$}$									
Non-exposed	192	1.00	Ref.		Non-exposed	192	1.00		
Tertile 1	7	1.02	0.48-2.18		Tertile 1	17	0.79	0.48-1.30	
Tertile 2	30	0.68	0.50-1.00		Tertile 2	17	0.66	0.40-1.08	
Tertile 3	15	0.81	0.47-1.40	0.535	Tertile 3	18	0.81	0.50-1.31	0.173
**Toxaphene**$^{\$}$									
Non-exposed	196	1.00	Ref.		Non-exposed	196	1.00	Ref.	
Tertile 1	10	2.15	1.14-4.08		Tertile 1	16	1.04	0.62-1.73	
Tertile 2	29	1.11	0.75-1.64		Tertile 2	17	1.56	0.94-2.57	
Tertile 3	12	1.19	0.65-2.17	0.712	Tertile 3	18	1.26	0.77-2.06	0.734
**Coumaphos**									
Non-exposed	405	1.00	Ref.		Non-exposed	405	1.00	Ref.	
Tertile 1	8	2.71	1.34-5.46		Tertile 1	12	1.05	0.59-1.87	
Tertile 2	22	1.05	0.69-1.62		Tertile 2	15	1.35	0.80-2.25	
Tertile 3	11	1.00	0.54-1.82	0.587	Tertile 3	14	1.14	0.67-1.95	0.552
**DDVP**									
Non-exposed	423	1.00	Ref.		Non-exposed	423	1.00	Ref.	
Tertile 1	7	2.18	1.03-4.59		Tertile 1	10	1.39	0.72-2.66	
Tertile 2	15	0.63	0.37-1.05		Tertile 2	11	0.56	0.31-1.03	
Tertile 3	10	0.89	0.47-1.67	0.855	Tertile 3	11	0.99	0.54-1.80	0.5987
**Captan**									
Non-exposed	412	1.00	Ref.		Non-exposed	412	1.00	Ref.	
Tertile 1	13	0.70	0.41-1.21		Tertile 1	12	0.72	0.41-1.27	
Tertile 2	15	1.07	0.58-1.96		Tertile 2	154	1.03	0.59-1.81	
Tertile 3	12	0.76	0.41-1.38	0.395	Tertile 3	154	0.79	0.44-1.42	0.301

Lifetime Days Pesticide Exposure					Intensity-Weighted Lifetime Exposure Days				
**Pesticide**	**Cases (n)**	**Hazard* Ratio**	**95% Confidence Interval**	**P for Trend**		**Cases (n)**	**Hazard* Ratio**	**95% Confidence Interval**	**P for trend**
**Benomyl[$]**									
Non-exposed	221	1.00	Ref.		Non-exposed	221	1.00	Ref.	
Q1	4	1.10	0.41-2.95		Q1	9	1.16	0.71-1.90	
Q2	10	1.00	0.53-1.90		Q2	6	1.37	0.77-2.44	
Q3	7	0.86	0.40-1.82		Q3	5	1.43	0.87-2.34	
Q4	4	1.25	0.46-3.41	0.003	Q4	5	0.76	0.41-1.39	0.659
**Chlorthalonil**									
Non-exposed	465	1.00	Ref.		Non-exposed	465	1.00	Ref.	
Q1	9	0.81	0.36-1.78		Q1	17	0.73	0.36-1.46	
Q2	24	1.53	1.00-2.32		Q2	15	0.80	0.45-1.44	
Q3	17	1.13	0.69-1.86		Q3	16	1.08	0.50-2.35	
Q4	10	0.97	0.49-1.92	0.873	Q4	12	0.83	0.42-1.62	0.932
**Metalaxyl[$]**									
Non-exposed	184	1.00	Ref.		Non-exposed	184	1.00	Ref.	
Q1	19	1.34	0.81-2.20		Q1	14	1.13	0.64-1.97	
Q2	16	1.02	0.61-1.71		Q2	16	1.20	0.70-2.05	
Q3	12	1.08	0.60-1.95		Q3	17	1.13	0.68-1.88	0.758
Q4	18	1.33	0.82-2.19	0.729	Q4	18	1.30	0.79-2.12	
**Methyl Bromide**									
Non-exposed	403	1.00	Ref.		Non-exposed	403	1.00	Ref.	
Q1	20	1.14	0.73-1.79		Q1	31	1.04	0.72-1.50	
Q2	44	1.01	0.74-1.37		Q2	27	0.89	0.60-1.31	
Q3	30	1.04	0.71-1.50		Q3	37	1.23	0.88-1.73	
Q4	28	1.01	0.68-1.49	0.432	Q4	26	1.01	0.67-1.52	0.706
**Ethylene Dibromide[$]**									
Non-exposed	228	1.00	Ref.		Non-exposed	228	1.00	Ref.	
Q1	3	0.67	0.21-2.07		Q1	5	0.95	0.39-2.30	
Q2	5	1.80	0.74-4.37		Q2	5	2.72	1.12-6.64	
Q3	4	18.8	6.85-51-58		Q3	6	2.03	0.90-4.58	
Q4	7	1.14	0.54-2.42	0.607	Q4	3	0.84	0.27-2.62	0.277

*Hazard ratios adjusted for age, smoking (pack-years among current and pack-years among former smokers) and gender, total lifetime pesticide use.

[$]Lifetime-days and Intensity-weighted lifetime Days of use were obtained from the take home questionnaire
